# Using a Support Vector Machine Based Decision Stage to Improve the Fault Diagnosis on Gearboxes

**DOI:** 10.1155/2019/1383752

**Published:** 2019-02-03

**Authors:** Rodrigo P. Monteiro, Mariela Cerrada, Diego R. Cabrera, René V. Sánchez, Carmelo J. A. Bastos-Filho

**Affiliations:** ^1^Federal University of Pernambuco, Recife 50740-550, Brazil; ^2^Universidad Politécnica Salesiana, Cuenca 010105, Ecuador; ^3^University of Pernambuco, Recife 50720-001, Brazil

## Abstract

Gearboxes are mechanical devices that play an essential role in several applications, e.g., the transmission of automotive vehicles. Their malfunctioning may result in economic losses and accidents, among others. The rise of powerful graphical processing units spreads the use of deep learning-based solutions to many problems, which includes the fault diagnosis on gearboxes. Those solutions usually require a significant amount of data, high computational power, and a long training process. The training of deep learning-based systems may not be feasible when GPUs are not available. This paper proposes a solution to reduce the training time of deep learning-based fault diagnosis systems without compromising their accuracy. The solution is based on the use of a decision stage to interpret all the probability outputs of a classifier whose output layer has the softmax activation function. Two classification algorithms were applied to perform the decision. We have reduced the training time by almost 80% without compromising the average accuracy of the fault diagnosis system.

## 1. Introduction

Gearboxes are mechanical devices that provide speed and torque conversion from rotating sources of power to other mechanisms. They have a crucial role in several applications, e.g., industrial rotating machines, automotive vehicles, and wind turbines. Their malfunction may not only impair the operation of a given system but also result in economic losses and safety risks [1]. This way, the use of fast and effective fault diagnosis techniques is necessary, since the early detection of failures allows more efficient management of the maintenance activities and leads to safer operation of the system [2].

Gearboxes may present several failure modes. Most of them are related to mechanical components and lubrication conditions. One failure mode that requires attention is the tooth breakage of gears, which is liable to compromise the machine operation in a significant way [3].

Supported by the advent of powerful computational devices, e.g., graphical processing units (GPUs), deep learning-based techniques have become essential tools in fault detection and fault diagnosis research fields. Their superior performance in applications related to classification and object detection tasks has also supported their popularization [4].

Plenty of works that relate deep learning and fault diagnosis in gearboxes have arisen in recent years. Zhao et al. [5] have proposed a variant of deep residual networks (DRNs) that uses dynamically weighted wavelet coefficients to improve the performance of the diagnostic process. Their work is based on the absence of a consensus about the most critical frequency bands regarding the useful information for systems that perform the diagnosis on planetary gearboxes. Their system finds discriminative sets of features by dynamically adjusting the weights applied to the wavelet packet coefficients. Cabrera et al. [6] proposed the use of a deep convolutional neural network (DCNN) trained in advanced by a stacked convolutional autoencoder (SCAE) to determine fault severity in gearboxes. Their system performs unsupervised detection of hierarchical time-frequency patterns using the DCNN. The SCAE improves the DCNN performance by capturing a priori patterns.

Furthermore, Deutsch and He [7] use a feedforward deep belief network (DBN) to predict the remaining useful life of mechanical machines. They combine the self-taught feature-learning capability of DBNs with the predicting power of feedforward neural networks to extract features from vibration signals, assess the integrity of the machine, and make the prediction. Jiang et al. [8] proposed the use of stacked multilevel-denoising autoencoders to perform the fault diagnosis on the gearboxes of wind turbines. The features are learned through an unsupervised process, which is followed by a supervised fine-tuning process with the label information for classification. They also use multiple noise levels to train the autoencoder and enhance the feature learning and classification capabilities. Jiang et al. [9] also proposed a gearbox diagnosis system based on multiscale convolutional neural networks. They combined multiscale and hierarchical learning to capture information at different scales, improving the performance of the classifier.

Monteiro et al. [10] proposed a fault diagnosis system based on the Fourier transform (FT) spectrograms and deep convolutional neural networks. They have also discussed in their work about the influence of the model depth and the amount of training data available on the network performance. Shao et al. [11] used transfer learning to perform the fault diagnosis on mechanical machines. A DCNN model pretrained on ImageNet, followed by a fine-tuning process, carried out the fault diagnosis. Other works, e.g., Zeng et al. [12] and Liao et al. [13], use convolutional neural networks associated with S and wavelet transforms to classify the gearbox heath condition, respectively.

One of the major issues about deep learning-based solutions for fault diagnosis systems is their computational burden; e.g., the training process of deep models is often long and demands a large amount of training data. Such a setback is usually overcome by using computers with powerful GPUs, e.g., [12]. However, this sort of hardware is not always available to everyone. Thus, it is necessary to find alternative ways to reduce the computational cost of deep learning-based solutions without compromising their performance regarding accuracy.

This paper proposes the addition of a decision stage in the output of DCNN-based fault diagnosis systems, which are commonly based on classification algorithms [5, 10, 12, 13]. The outputs of those systems often represent the probabilities of a given input to belong to a failure mode in a given set. The failure mode that presents the highest probability value is chosen. Although this approach has proved to be reasonable to a number of applications, the information provided by the remaining outputs is usually lost.

We believe that this information can also be used to improve the performance of the classifier. The case study is the one analyzed in [6, 7], which poses the problem of the fault severity diagnosis related to the gear tooth break failure mode. The decision stage analyses the outputs of all classes, i.e., severity levels, and decides the severity of the gearbox fault based on their probabilities distribution. Since this artifice improves the classification results, the same model architecture can be trained within fewer epochs without compromising its accuracy, thus reducing the training time. Decision stages, on the other hand, are well-known tools. They are commonly employed in multimodal and committee-based classification systems. They combine the results obtained by multiple classifiers to improve the accuracy of the whole system [14, 15].

The remainder of this paper is defined as follows: Section 3 presents the details of the experiments carried out in this research, Section 4 presents the results obtained and discusses their relevance, and Section 5 explains the main findings and implications of this work.

## 2. Theoretical Background

### 2.1. Convolutional Neural Networks

**Figure 1:**
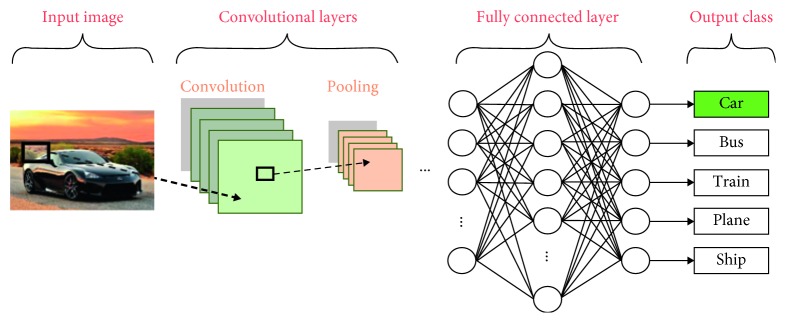
Basic structure of a convolutional neural network [16].

The convolutional neural networks are models inspired by biological processes. The pattern of the connections among neurons, i.e., the processing units of neural networks, is similar to that of the animal visual cortex. They perform object recognition and classification tasks [16] well. Object detection [17], diseases detection [18], and fault diagnosis [6, 10] are three examples of applications that use CNNs. Their basic structure consists of an input layer, alternating blocks of convolutional and pooling layers, which are followed by fully connected layers, and an output layer [16]. Modifications in this structure may occur, depending on the application. This structure is illustrated in Figure 1. The role of each layer is explained as follows:

(a) Input layer: this layer receives and stores raw input data. It also specifies the width, height, and number of channels of the input data [19].
(b) Convolutional layers: they learn feature representations from a set of input data and generate feature maps. Those maps are created by convolving their inputs with a set of learned weights. An activation function, e.g., the ReLU function, is applied to the output of the convolution step. The following equation shows the general formulation of a convolutional layer:
  (1) $$\begin{array}{c} x_{j}^{l}=f\left(\underset{i\in M_{j}}{\sum }x_{i}^{l-1}*k_{ji}^{l}+b_{j}^{l}\right), \end{array}$$
  in which *l* refers to the current layer, *i* and *j* are the indices of the elements of the previous and current layers, respectively, *M*_*j*_ is a set of input maps, *k* is the weight matrix of the *i*-th convolutional kernel of the *l*-th layer applied to the *j*-th input feature map, and *b* is the bias.
(c) Pooling layers: they reduce the spatial resolution of feature maps, improving the spatial invariance to input distortions and translations [19]. Most of the recent works employ a variation of this layer called the max pooling [16]. It propagates to the next layers the maximum value from a neighborhood of elements. This operation is defined by
  (2) $$\begin{array}{c} y_{j\text{rs}}=\underset{\left(p,q\right)\in R_{\text{rs}}}{\max}x_{kpq}, \end{array}$$
  in which *y*_*j*rs_ is the output of the pooling process regarding the *j*-th feature map and *x*_*kpq*_ is the element at location (*p*; *q*) contained by the pooling region *R*_rs_. The pooling process is also known as subsampling [19].
(d) Fully connected and output layers: they interpret feature representations and perform high-level reasoning [16]. They also compute the scores of each output class [19]. The number of output nodes depends on the number of classes [12].

### 2.2. Fourier Transform Spectrograms

The Fourier transform (FT) is an essential technique in the field of signal analysis. It informs the frequency composition of a given signal, as well as the contribution of each frequency concerning magnitude [20]. Noise filtering, pattern recognition, and signal modulation are some applications that may be improved by the Fourier transform and its variants, e.g., the discrete Fourier transform (DFT), suitable for processing digital signals, and the fast Fourier transform (FFT), a more efficient algorithm to calculate the DFT [21].

The Fourier transform spectrograms represent signals using time, frequency, and magnitude information. The short-time Fourier transform (STFT) is an FT variant commonly used to generate this sort of representation because it performs time-dependent spectral analyses [21]. The spectrograms show how the spectrum of frequencies of a given signal varies over time. Spectrograms are also used in fault diagnosis applications [10, 22].

### 2.3. Support Vector Machines

The support vector machine (SVM) is a versatile and powerful machine learning technique [23]. It can be used to solve classification (both linear and nonlinear), regression, and even outlier detection problems, making it one of the most popular machine learning algorithms [23, 24]. Its use is also popular in fault diagnosis of rotating machinery [25]. This technique aims the identification of hyperplanes capable of separating datasets into high-dimensional feature spaces. The separation between datasets is called margin, and the SVM maximizes the margin [23].

A linearly separable dataset allows the SVM to define hyperplanes capable of separating the data into categories, regardless of the number of dimensions presented by the feature space. However, in most applications, the information is not linearly separable in feature spaces with a given dimensionality. Thus, it is necessary to map the dataset into a feature space with a higher number of dimensions, in which the data will be linearly separable. This mapping process is performed by using kernels, e.g., polynomial and radial basis function kernels [23, 24].

### 2.4. Multilayer Perceptron

The multilayer perceptron (MLP) is a feedforward neural network. MLPs can distinguish nonlinearly separable patterns. Those algorithms consist of several nodes, named “neurons,” which are arranged in multiple layers just as a directed graph. Each layer is fully connected to the subsequent one. Those layers are usually divided into three types: input, hidden, and output layers. Multilayer perceptrons are considered to be universal approximators. One hidden layer MLP with enough neurons can approximate any given continuous function [23, 24].

## 3. Materials and Methods

### 3.1. Experimental Setup: Obtaining the Vibration Signals

**Figure 2:**
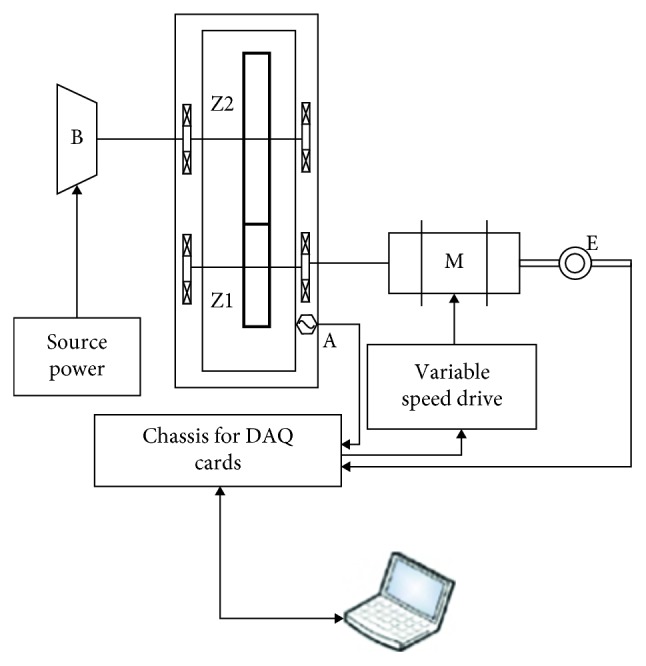
Experimental setup [10]. A, accelerometer; B, break system; M, motor; E, encoder; Z1, first gear; Z2, second gear.

We arranged the experimental setup according to Figure 2. It was used to obtain the vibration measurements of the gearbox. The electric motor (M) drives the gearbox, composed of two gears (Z1 and Z2). Those gears are mounted on independent shafts. A magnetic brake (B) is connected to the output shaft.  Table 1 lists some features of those components.

Besides, the speed drive Danfoss VLT 1 : 5 kW drives the electric motor, and the voltage source power TDK Lambda (GEN 150-10, 0–150 V, 10 A) drives the magnetic brake. A unidirectional accelerometer (*A*), which was vertically placed on the gearbox, close to the input shaft, collects the vibration signals. This accelerometer is an IMI Sensor 603C01, 100 mV/g. An NI9234 data acquisition card performs the digitalization of analog signals. This card has a 24-bit resolution, a 50 kHz sampling rate, and it is specific for piezoelectric sensors.

**Figure 3:**
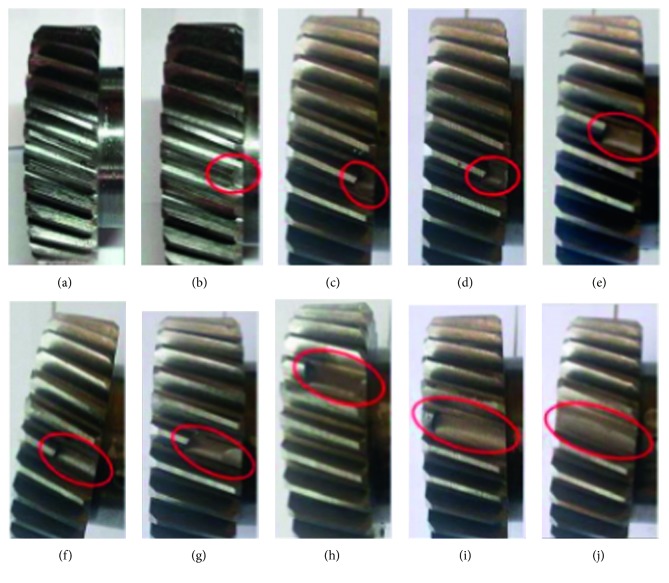
Ten scenarios of gear tooth breakage. (a) Class P1. (b) Class P2. (c) Class P3. (d) Class P4. (e) Class P5. (f) Class P6. (g) Class P7. (h) Class P8. (i) Class P9. (j) Class P10.

As previously mentioned, the proposed experiment aims to use the vibration signals of the gearbox to evaluate the severity of tooth breakage faults in helical gears. For this purpose, one tooth of the helical gear Z1 was subjected to different damage levels. On the other hand, gear Z2 has not been modified. Ten scenarios were taken into account, i.e., one for the gear Z1 unbroken and the others regarding nine fault severity levels of gear Z1. Those scenarios are listed in Figure 3 and Table 2.

We have also considered the gearbox working under different operation conditions; that is, we took into account different loads and rotation speeds. The rotation speed had five scenarios, in which it was constant in three of them and variable in the others. On the other hand, the load applied by the magnetic braking system had three scenarios, in which the load has presented constant values. Those scenarios are detailed in Tables 3 and 4, for the rotation speeds and the loads, respectively.

We acquired each sample of vibration signal over a time interval of 10 s. Furthermore, we performed each combined scenario three times. This way, the database is composed originally by 45 signals for each fault severity, that is, a balanced database of 450 signals considering all the ten severity levels. The magnitude of those signals was normalized to the range [0, 1] and divided into 0.25 seconds lengthy excerpts, resulting in 1,800 signals for each fault severity level and 18,000 signals in the whole and balanced database.

### 3.2. Experimental Setup: Training the Classification System

The system proposed to assess the fault severity of the gearbox is based on a deep convolutional neural network architecture. Thus, a bidimensional representation of the input signals was necessary. We chose to represent them on a time-frequency domain, since this sort of representation allows to visualize when the specific frequency components related to the failure arise.

The short-time Fourier transform was the technique we used to generate the bidimensional representation of the signals, i.e., the Fourier transform spectrograms. The STFT has low computational cost than other time-frequency representation techniques [26]. This characteristic is especially important to the proposed system since we are dealing with a real-time application. The STFT configuration included a Hamming window of size 128 and overlapping equal to 50%. These choices combined the selective property of the Hamming window with the balance between the smooth variation of the resulting signal and low computational cost.

**Figure 4:**
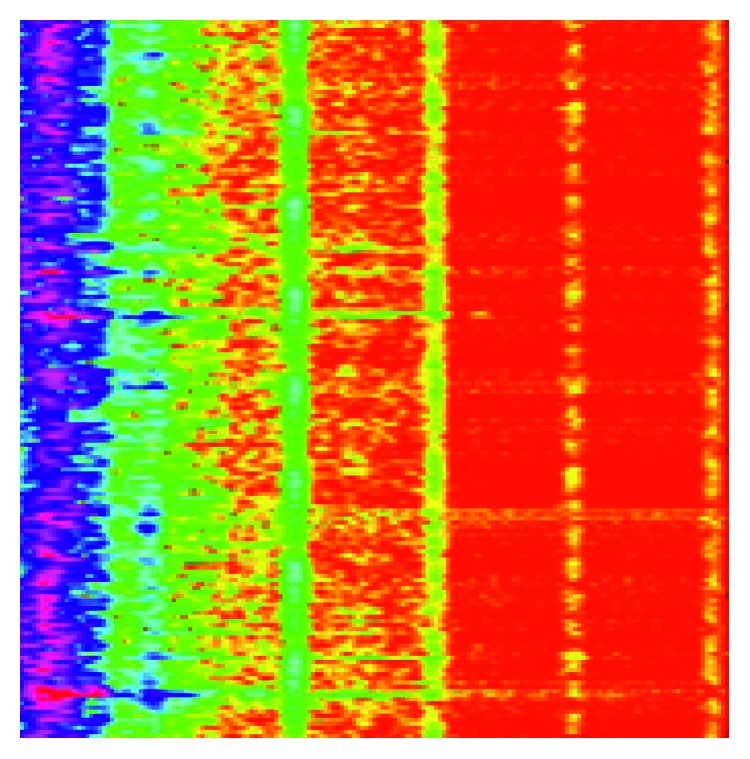
Spectrogram obtained by using the STFT.

Two experimental scenarios were designed. In the first one, the signal information was condensed into 175 × 175 pixels RGB images. This sort of data allows more information to be provided to the fault severity assessment system, since artifices such as colormaps can be used. On the other hand, it increases the computational burden of the system because the input presents 3 channels. In the second scenario, we used 175 × 175 pixels grayscale images. Unlike the previous scenario, the only information provided by spectrograms is the magnitude of the Fourier transform.  Figure 4 shows an example of spectrogram obtained by the described process.

**Figure 5:**
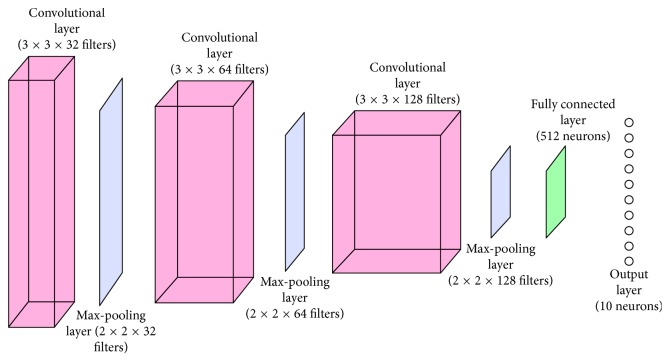
Network architecture.

The classification system used in this work is composed of three convolutional layers, three max-pooling layers, one fully connected layer, and one output layer. Since the outputs of such structure are probability values in the range between 0 and 1, the softmax activation function was used in the neurons of the output layer and the ReLU activation function was used in the neurons of the remaining layers. This architecture provided a satisfactory performance in the fault severity assessment of gearboxes, according to Monteiro et al. [10]. It is illustrated in Figure 5. In addition, a support vector machine was used to analyze the output of the system and improve its performance. This algorithm was already used in similar applications, like the one proposed by Li et al. [27], which employed the SVM to merge the results of classifiers that act on multimodal data.

Regarding the training step, the data were split into three groups: training, test, and validation. We used the validation dataset to reduce the occurrence of overfitting-related problems. Each one contains, respectively, 50%, 25%, and 25% of balanced signals for each fault severity level. The training process was performed according to 10 and 50 epoch scenarios. The configuration of the computer used to train the model was OS Windows 10 Home, 64 bits, memory (RAM) 15.9 GB, processor Intel® Core™ i7-6500 CPU @ 2.50 GHz × 2, and AMD Radeon™ T5 M330 (No CUDA support).

## 4. Results and Discussion

The first discussion is regarding the training time of a fault diagnosis system based on deep convolutional neural networks. It is well known that computers with GPUs can handle the computational burden of deep learning solutions much better than those with CPUs. On the other hand, computers with GPUs are more expensive, meaning that they are not always available.  Table 5 illustrates this problem. It shows the average training time of 30 DCNNs (in each scenario) with the architecture mentioned in the last section, regarding computers with different configurations and the RGB image dataset. The models were trained in 50 epochs. We trained this number of models to guarantee the statistical relevance of the results. The first computer configuration (GPU computer) was used by Monteiro et al. [10]. It consisted of a OS Ubuntu 16.04 LTS, 64 bits, memory 15.6 GB, processor Intel Xeon(R) CPU E 5-2609 v3 @ 1.90 GHz × 12, and graphics Gallium 0.4 on NV117. The second one was presented in the previous section.

One can observe that the training process for the computer without the GPU was much longer than the one for the computer with GPU; i.e., it was about 13 times longer. In some situations, depending on the amount of data or time available, the use of computers without GPUs can be impractical.

Some choices can be made to overcome this problem. Reducing the number of training epochs is one of them. As can be observed in Table 6, reducing the number of training epochs from 50 to 10 decreased the average training time in about 78.7%. On the other hand, such reduction had a performance cost. The average accuracy reduced about 1.8%. This behavior was already expected, since the models had less iterations to learn features of the training data. The results in Table 6 were obtained by training 30 DCNNs in each scenario.

Before proposing modifications to the fault diagnosis system, it is necessary to identify the major difficulties of the model. We have taken the model trained for 10 epochs as the reference.  Table 7 lists the average and standard deviation values of the accuracy for 30 models. One can observe that for some classes, e.g., P1 and P2, the system presented high accuracy values, i.e., close to 100%. On the other hand, the models presented a poor performance for the input data from classes such as P6 and P7.

**Figure 6:**
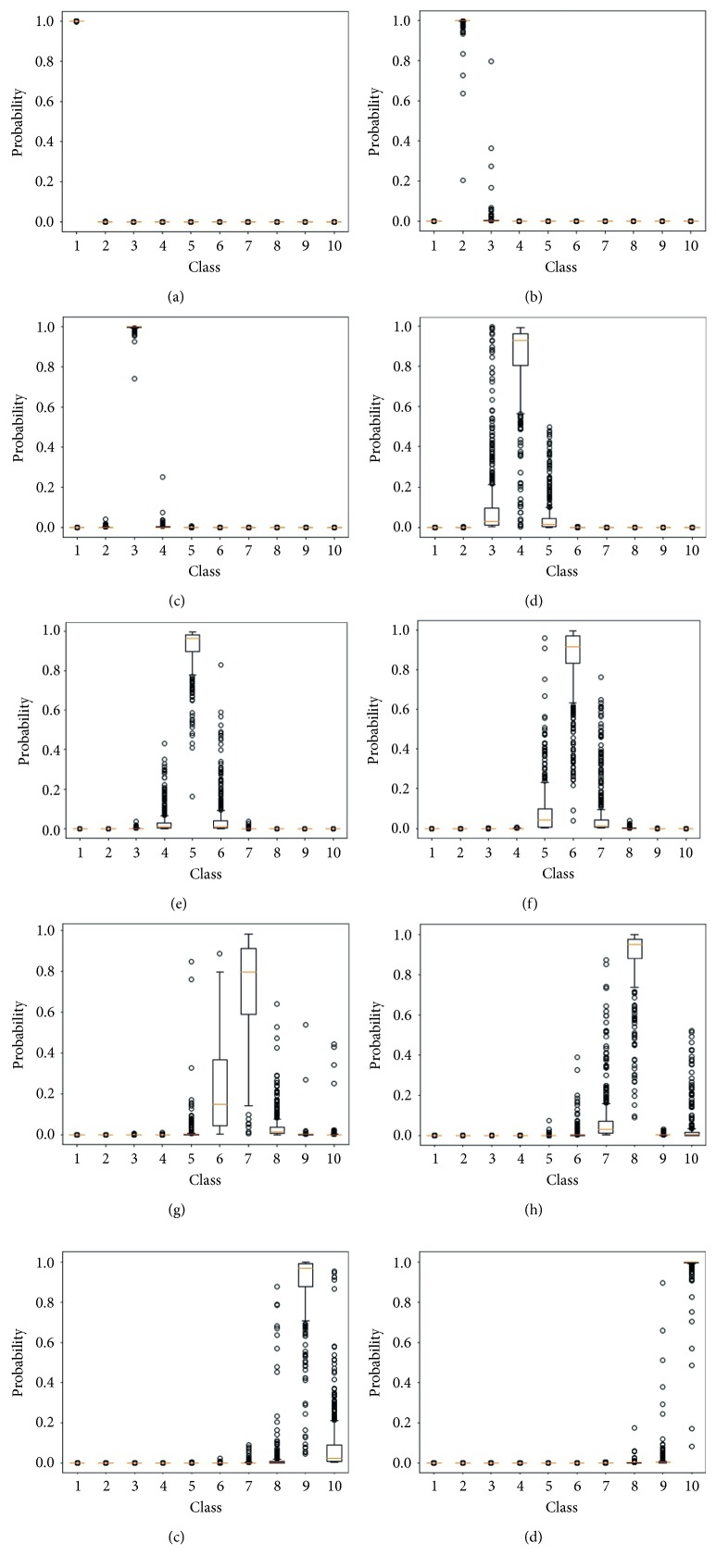
Output probabilities of models trained for 10 epochs, for all the ten classes. (a) Class P1. (b) Class P2. (c) Class P3. (d) Class P4. (e) Class P5. (f) Class P6. (g) Class P7. (h) Class P8. (i) Class P9. (j) Class P10.

This analysis can be deepened by observing the outputs of the classifier.  Figure 6 shows how the output probabilities of the models are distributed according to the class of the input image. Regarding the inputs belonging to class P1, one can observe that the output probabilities of the models were very close 1 for class P1 and very close to 0 for the others. It helps to understand why the model accuracy for this class was 100%. On the other hand, the distribution profiles belonging to classes P6 and P7 show that the outputs of the networks were not so accurate as in the previous case. Indeed, choosing only the output that presented the highest probability value can lead to wrong classifications due to the significant presence of outliers.

To overcome this issue, we proposed a solution based on using the output probabilities of all the ten classes to perform the correct classification. Such a solution can be implemented in several ways. One of them is the use of shallow classifiers, e.g., multilayer perceptron or support vector machine. Those classifiers identify the gear severity class by using the information contained in the output probabilities of the deep convolutional neural network. Thus, the system response is obtained by analyzing a probability distribution, and not only a single value.

We used the support vector machine in this research. It was trained with the outputs of the DCNN regarding the training data previously established. The results of the proposed modification are listed in Tables 8 and 9.  Table 8 shows the average results of each class, regarding 30 models, and compares them with the results of the scenario without the additional classifier.  Table 9 shows the average results regarding all classes and models, also comparing to the scenario without the additional classifier.

From Table 8, one can infer that the inclusion of a classifier improved the model performance regarding all the 10 classes, both concerning average accuracy and standard deviation. Also, from Table 9, one can observe that the average accuracy increased about 2.56% by the cost of increasing less than 1 second to the average training time. These results are even more significant when compared with those obtained from the training process with 50 epochs, seen in Table 5. The average accuracy of the proposed model was only 0.76% higher, but with an average training time 78.64% smaller. This made the training of the fault diagnosis system significantly faster. Furthermore, we employed two additional metrics to ensure the reliability of the obtained results: the *F*-score and AUC. The first one is the harmonic mean of the precision and recall. The second metric, on the other hand, is defined as the area under the curve of the receiver operating characteristic (ROC). Their average values are listed in Table 10, and both of them show an improvement trend aligned to the one observed in Table 9, i.e., the diagnosis system with the classifier presented values for the metrics about 2% higher than that without the classifier.

Regarding the average time to perform the classification of one single input, the addition of a decision stage did not cause significant changes. Indeed, the average classification time, which was about 0.03 seconds without the decision stage, increased less than 0.001 seconds.

To evaluate how significant were the improvements provided by the proposed solution, the signal-ranked Wilcoxon statistical test can be applied to the outputs of the fault diagnosis systems with and without the decision stage. The results are listed in Table 10. The Wilcoxon test is a nonparametric hypothesis test that can be used to assess if two distributions are equivalent or not [28]. If they are not, it means that there was a statistically significant improvement (Λ symbol). Otherwise, the improvement was not significant (— symbol).  Table 11 shows that we had significant improvements for classes P4, P5, P6, P7, and P9. Although the remainder classes have also shown some improvement, they were not statistically significant.

We also analyzed the performance of the decision stage when a different classification algorithm was applied. This algorithm was the multilayer perceptron, i.e., a neural network. We set the size of the input layer equal to 10, the hidden layer with 21 neurons, and the output layer with one neuron. We deployed the logistic sigmoid as the activation function. We set the number of training epochs equal to 200. The number of training, test, and validation samples remained the same as in the SVM scenario. We trained 30 MLPs to guarantee the statistical relevance of the results. We show the results for the SVM and MLP in Tables 12 and 13.  Table 12 lists the average values of *F*1-score, AUC, and accuracy.  Table 13 lists the average training and running times.

Regarding the metrics, despite the slight advantage presented by the SVM decision stage concerning the accuracy, a Wilcoxon test suggested that the results achieved by both algorithms were the same. It means that the proposed solution can be implemented with other classifiers than the SVM. On the other hand, an interesting fact arises from the training and running times. The training process of the MLP decision stage was about 7.1 times longer than that of the SVM, whereas the running time was more than 20 times faster. The proper decision considering this trade-off may be attractive depending on the contemplated application.

Those analyzes with the SVM decision stage were also performed regarding the grayscale spectrogram images. They aimed to evaluate how the fault diagnosis system would deal with the reduction of the available information.  Table 14 shows the average accuracy and the average training time for 30 models belonging to each scenario. These scenarios regarded models trained with RGB and grayscale images, for 10 epochs. Only the test data were employed in the calculation of the average accuracy. One observed that a model trained with RGB images provided the best results regarding accuracy, i.e., about 10% higher. On the other hand, the average training time of the models trained with grayscale images was lower. It probably happened due to the smaller amount of information that was processed.

**Figure 7:**
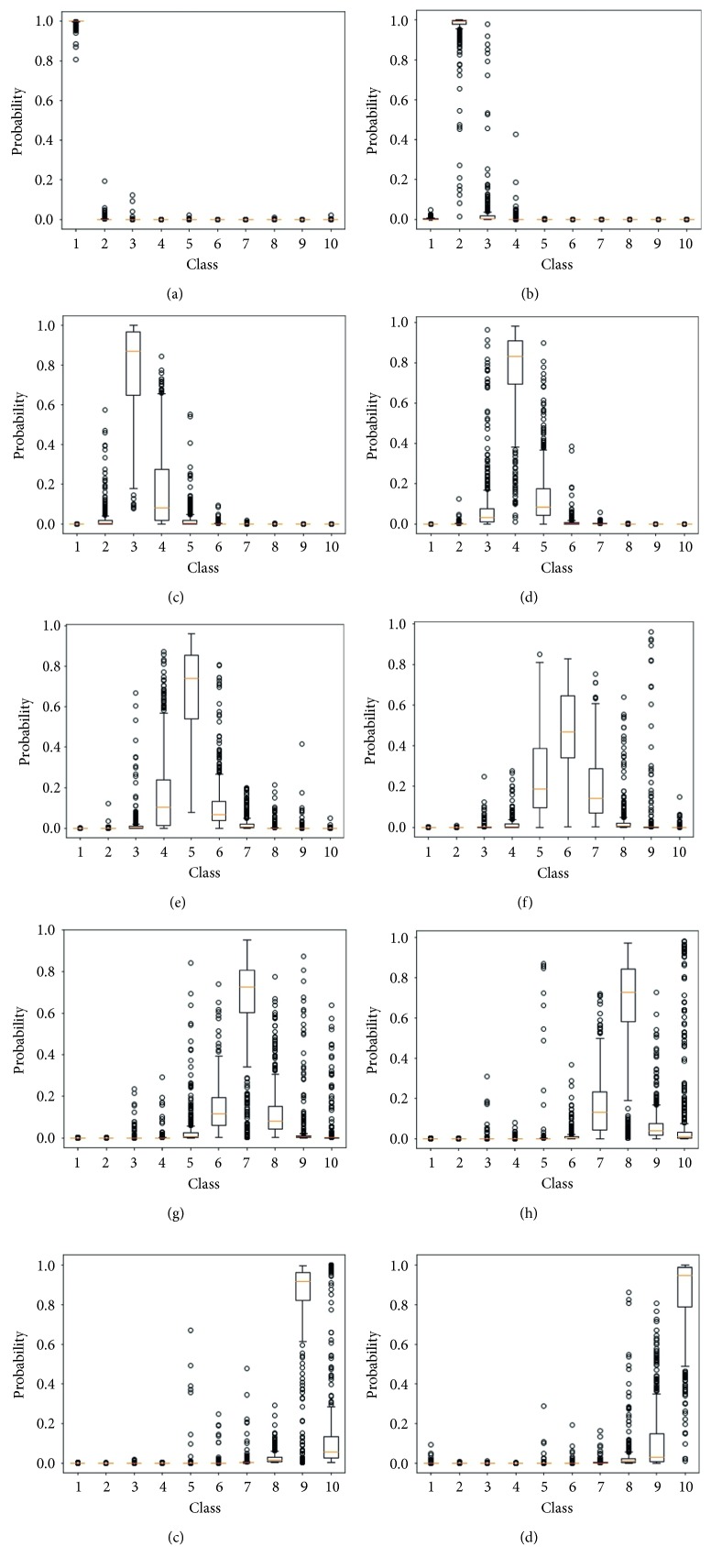
Output probabilities of models trained for 10 epochs using grayscale images, for all the ten classes. (a) Class P1. (b) Class P2. (c) Class P3. (d) Class P4. (e) Class P5. (f) Class P6. (g) Class P7. (h) Class P8. (i) Class P9. (j) Class P10.

Table 15 and Figure 7 help to understand what is happening to the performance of the classification model trained using the grayscale images, regarding all the ten classes. One can observe that, concerning the RGB scenario, even the results of classes like P1 were worsened, for both average accuracy and standard deviation. Figure 7 shows how the output probabilities of the DCNN are distributed according to the class of the input image. The number of outliers has significantly increased for the other scenario. This behavior occurred to all classes.

Also, employing the decision stage in this new scenario, we intend to evaluate if the proposed solution can improve the performance of the DCNN-based classifier by using the output probabilities of all the 10 classes.

The results obtained by using a support vector machine trained with the outputs of the deep convolutional neural networks are listed in Tables 16 and 17.  Table 16 shows the average results for each class regarding 30 trained models. Those results are compared with those of the scenario without the additional classifier.  Table 17 shows the average results regarding all classes and models, also comparing them with the scenario without the additional stage. From Table 16, one can infer that the inclusion of a classifier improved the model performance regarding all the ten classes, both regarding average accuracy and standard deviation. On the other hand, even these improved systems were not capable of outperforming those trained with the RGB spectrogram images, as seen in Table 14. Besides, from Table 17 one can observe that the average accuracy increased about 4.18% by the cost of increasing less than 1 second to the average training time. This relative improvement was superior to the one observed in the previous experimental scenario.  Table 18 shows the average *F*-score and AUC for the systems with and without the decision stage.  Table 18 shows the average *F*-score and AUC for the systems with and without the decision stage. These two metrics reinforce the improvement trend caused by the addition of a decision stage.

Regarding the average classification time, in this scenario, the addition of a decision stage did not perform significant changes as well. It increased from 0.022 seconds to less than 0.023 seconds.

To evaluate how significant was the improvement provided by the proposed solution in this new scenario, the signal-ranked Wilcoxon statistical test was also applied on the outputs of the fault diagnosis systems with and without the decision stage. The results are listed in Table 19, which show that we had significant improvements for classes P4, P5, P6, P7, P8, and P10. Once again, although the other classes have also shown improvements, they were not significant.

## 5. Conclusions

We analyzed the use of a decision stage to interpret the outputs of a fault diagnosis system based on deep convolutional neural networks. Those outputs correspond to the probability that an input belongs to the classes of a given set. This way, instead of using a conventional approach like choosing the class with the highest probability value, we analyzed the output distribution of the deep classifier to perform more reliable fault diagnosis.

The results have shown that we could improve the accuracy of the classification system and reduce almost 80% of the training time without compromising the execution time, which increased about 0.001 seconds. This improvement is especially significant for situations in which the powerful hardware, e.g., graphical processing units, is not available. Thus, a fault diagnosis system with a given accuracy value can be obtained by using only a small fraction of the training time that would be required to perform the complete training. Those results were achieved by using the SVM as the decision maker, which had the output probabilities of the original fault diagnosis system as input information. Similar results were achieved by implementing an MLP as the decision maker. It suggests that the proposed solution can also be implemented with algorithms other than the SVM.

We also assessed the use of RGB and grayscale input spectrograms. Although the addition of a decision stage caused improvements in both scenarios, these improvements presented different magnitudes. The accuracy of systems operating on grayscale images increased more than that in the other scenario. However, the final accuracy of those systems trained on RGB images was superior. This behavior can be explained by the amount of information available in each kind of image, as previously discussed. Furthermore, we saw that the difference presented by the execution times in both scenarios was not significant concerning absolute values. Thus, it suggests that using RGB images would not compromise the operation of the system on real-time applications.

Future works regard the application of this methodology of fault diagnosis to other kinds of failures and for problems belonging to different physical domains, e.g., fault diagnosis using acoustics. Moreover, we can evaluate the performance of other algorithms employed as decision-makers.

## Figures and Tables

**Table 1:** Characteristics of the components of the experimental arrangement.

Component	Description
Electric motor (M)	Motor Siemens 1LA7 090-4YA60 1 : 49 kW, 4 poles, and 28 : 33 Hz
Gear 1 (Z1)	Pinion: 76 mm, 30 teeth, pressure angle = 20, and helix angle = 20
Gear 2 (Z2)	Gear: 112 mm, 45 teeth, pressure angle = 20, and helix angle = 20
Brake (B)	Magnetic brake: proportional force to input voltage and belt coupled

**Table 2:** Damage severity levels of gear Z1 tooth breakage fault.

Code	Damage (mm)	Tooth percentage (%)
P1	0.00	100.00
P2	2.37	88.42
P3	4.00	80.42
P4	5.73	71.94
P5	7.60	62.81
P6	10.57	48.29
P7	12.37	39.48
P8	14.33	29.85
P9	17.15	14.36
P10	20.43	0.00

**Table 3:** Rotation frequencies.

Code	Frequency profile	Rotation frequency (Hz)
F1	Constant	8
F2	Constant	12
F3	Constant	15
F4	Sine period = 2 s	8–15
F5	Square period = 2 s	8–15

**Table 4:** Loads.

Code	Voltage supplied to the magnetic brake
L1	0 V
L2	10 V
L3	30 V

**Table 5:** Average training time of DCNNs with different computer configurations.

Configuration	Average training time (s)
Computer with GPU	815
Computer with CPU	10,601

**Table 6:** Average accuracy and average training time for models trained with 50 and 10 epochs.

Number of training epochs	Average accuracy (%)	Average training time (s)
50	96.64	10,601
10	94.84	2,263

**Table 7:** Average accuracy and standard deviation of 30 models trained for 10 epochs.

Class	Average accuracy (%)	Standard deviation
P1	100	0
P2	99.79	0.32
P3	98.30	6.20
P4	95.92	4.47
P5	94.32	7.66
P6	89.89	12.30
P7	83.79	13.03
P8	94.10	7.86
P9	96.50	4.60
P10	95.87	7.26

**Table 8:** Average accuracy and standard deviation of models that present or not an additional classifier.

Class	Without additional classifier		With additional classifier	
	Average accuracy (%)	Standard deviation	Average accuracy (%)	Standard deviation
P1	100	0	100	0
P2	99.79	0.32	99.79	0.32
P3	98.30	6.20	99.42	0.79
P4	95.92	4.47	97.74	1.71
P5	94.32	7.66	97.56	1.89
P6	89.89	12.30	94.36	4.29
P7	83.79	13.03	92.70	3.36
P8	94.10	7.86	96.71	1.84
P9	96.50	4.60	97.78	1.66
P10	95.87	7.26	97.96	1.51

**Table 9:** Average accuracy and average training time for models that present or not an additional classifier.

	Average accuracy (%)	Average training time (s)
Without classifier	94.85	2,263
With classifier	97.4	2,264

**Table 10:** Average *F*-score and average AUC for models that present or not an additional classifier.

	Average *F*-score	Average AUC
Without classifier	0.95	0.97
With classifier	0.97	0.99

**Table 11:** Results of the signal-ranked Wilcoxon test comparing the results with and without the output layer for the decision-making process.

Class	Wilcoxon result
P1	—
P2	—
P3	—
P4	Λ4
P5	Λ5
P6	Λ6
P7	Λ7
P8	—
P9	Λ9
P10	—

**Table 12:** Average *F*-score, AUC, and accuracy for models that present MLPs and SVMs as decision stages.

	Average *F*-score	Average AUC	Average accuracy (%)
SVM	0.97	0.99	97.4
MLP	0.97	0.99	97.25

**Table 13:** Average training and running times for models that present MLPs and SVMs as decision stages.

	Average training time (s)	Average running time
SVM	0.33	0.00022
MLP	2.35	0.00001

**Table 14:** Average accuracy and average training time for models trained with RGB and grayscale.

Scenario	Average accuracy (%)	Average training time (s)
Grayscale	84.98	1,967
RGB	94.84	2,263

**Table 15:** Average accuracy and standard deviation of models trained for 10 epochs using grayscale images.

Class	Average accuracy (%)	Standard deviation
P1	99.98	0.07
P2	98.35	1.33
P3	93.04	6.96
P4	81.58	12.42
P5	75.50	15.05
P6	69.13	19.51
P7	74.30	15.03
P8	82.70	7.16
P9	85.47	8.51
P10	89.81	9.57

**Table 16:** Average accuracy and standard deviation of models that present or not an additional classifier.

Class	Without additional classifier		With additional classifier	
	Average accuracy (%)	Standard deviation	Average accuracy (%)	Standard deviation
P1	99.98	0.07	9.99	0.06
P2	98.35	1.33	9.50	0.65
P3	93.04	6.96	94.73	1.78
P4	81.58	12.42	86.64	3.82
P5	75.50	15.05	81.26	3.54
P6	69.13	19.51	79.60	4.14
P7	74.30	15.03	81.61	2.47
P8	82.70	7.16	86.16	1.83
P9	85.47	8.51	88.46	1.41
P10	89.81	9.57	94.70	2.89

**Table 17:** Average accuracy and average training time for models that present or not an additional classifier.

	Average accuracy (%)	Average training time (s)
Without classifier	84.98	1,967
With classifier	89.16	1,968

**Table 18:** Average *F*-score and average AUC for models that present or not an additional classifier.

	Average *F*-score	Average AUC
Without classifier	0.85	0.92
With classifier	0.89	0.94

**Table 19:** Wilcoxon test.

Class	Wilcoxon result
P1	—
P2	—
P3	—
P4	Λ4
P5	Λ5
P6	Λ6
P7	Λ7
P8	Λ8
P9	—
P10	Λ1

## Data Availability

The data used to support the results of this study are included within the article.
